# Computational Insights into Novel Inhibitor *N*-(3-(*tert*-Butylcarbamoyl)-4-methoxyphenyl)-indole and Ingliforib Specific against GP Isoenzyme Dimers Interaction Mechanism

**DOI:** 10.3390/molecules28134909

**Published:** 2023-06-22

**Authors:** Youde Wang, Shuai Li, Zhiwei Yan, Liying Zhang

**Affiliations:** Laboratory of Traditional Chinese Medicine Research and Development of Hebei Province, Institute of Traditional Chinese Medicine, Chengde Medical University, Chengde 067000, China

**Keywords:** glycogen phosphorylase, *N*-(3-(*tert*-butylcarbamoyl)-4-methoxyphenyl)-indole, GP subtype molecular docking, molecular dynamics, ingliforib

## Abstract

The high conservation of the three subtypes of glycogen phosphorylase (GP) presents significant challenges for specific inhibitor studies targeting GP. Our prior screening revealed that compound **1** exhibited unequal inhibitory activity against the three GP subtypes, with a noticeable effect against brain GP (PYGB). The commercially available ingliforib demonstrated potent inhibitory activity specifically against liver GP (PYGL). To guide the further design and screening of high-specificity inhibitors, the possible reasons for the differential inhibitory activity of two compounds against different GP subtypes were analyzed, with ingliforib as a reference, through molecular docking and molecular dynamics simulations. Initially, the study predicted the binding modes of ligands with the three GP receptor subtypes using molecular docking. Subsequently, this was validated by molecular dynamics experiments, and possible amino acid residues that had important interactions were explored. The strong correlation between the calculated interaction free energies and experimental inhibitory activity implied the reasonable binding conformations of the compounds. These findings offer insight into the different inhibitory activity of compound **1** and ingliforib against all three GP subtypes and provide guidance for the design of specific target molecules that regulate subtype selectivity.

## 1. Introduction

**Figure 1:**
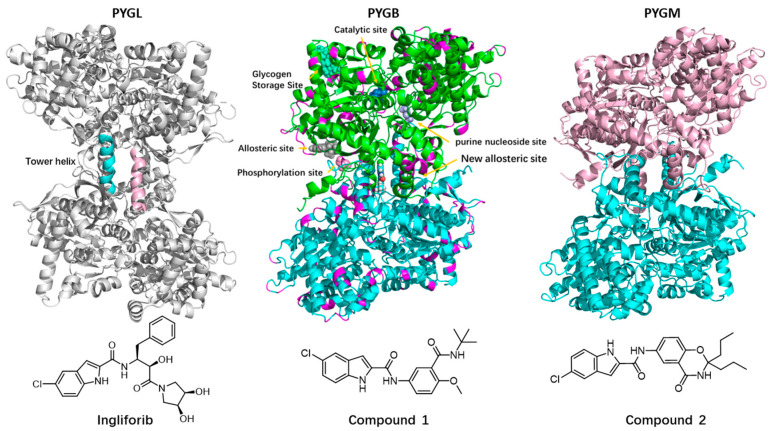
X-ray structures of three GP subtypes showing the binding sites and chemical structure of compound **1**. The protein is displayed through in cartoon fashion, and the inhibitor is displayed through the sphere model. The purple part in the crystal structure indicates the difference in amino acids between PYGB and the other two subtypes.

Glycogen phosphorylase (GP) is a critical rate-limiting enzyme that catalyzes the breakdown of glycogen into glucose-1-phosphate during glycogenolysis. This reaction is vital in maintaining glucose homeostasis, which is necessary to sustain normal cellular function. Therefore, GP was initially considered a potential target for the treatment of type 2 diabetes (T2D) [1]. Recently, GP inhibitors have also been found to have a positive effect on myocardial ischemia, cerebral ischemia and cancer [2,3,4]. In mammals, GP is a family of three isozymes, named brain GP (PYGB), liver GP (PYGL) and muscle GP (PYGM), according to the tissue in which they are expressed, and they exist as active or inactive homodimers (Figure 1) [5]. Studies have shown that the different GP isoforms usually prioritize different types of physiological activity; for example, the inhibition of PYGL lowers blood glucose [6] and the inhibition of PYGB facilitates myocardial protection [7], while PYGM is associated with muscle GP deficiency (McArdle disease), schizophrenia and cancer [8]. However, these isozymes were found to be 80% homologous [9], and cross-reactivity may occur when non-selective GP inhibitors are used [10]. Thus, one of the challenges in developing inhibitors of GP is to achieve sufficient selectivity for enzyme isoforms.

With advances in structural biology and computational modeling providing new insights into the mechanisms of GP inhibition, this could inform the design of new inhibitors with better potency and selectivity. The crystal structure of PYGM was first resolved in the early 1970s [11]. Many structures of this isoenzyme were then obtained in the presence of different metastable effectors and/or drugs to better understand the metastable regulation of PYGM. Later, in 2000, the crystallographic structure determination of PYGL revealed the structural basis for the differences in the regulation of these isozymes [12]. In 2016, the crystal structure of human PYGB was obtained for the first time by Mathieu et al. (PDB ID: 5IKO) [13]. Comparison of the structures of the three isozymes showed that the structure of PYGB is highly similar to the active state of PYGM [14]. This may be the reason that our previous compound **2**, with its IC_50_ value for PYGB, was closer to that of PYGM compared to the IC_50_ value for PYGL [3]. Meanwhile, they also show some differences, such as the tower helix (helix 7 of each monomer), which is a major component of the dimerization interface in GP isozymes, and these two helices show antiparallel binding and control the dimerization and activation of the enzyme. They show typical crossover angles of 75°, 84° and 45° in active PYGB, PYGM and PYGL, respectively [14]. Freeman et al. explored the potential selectivity of GPi688 and GPi819 against the liver and muscle GP isoforms and showed that the sensitivity of the inhibitors was dependent on the activation state of the enzyme [15]. To avoid cross-reactivity between GP isoforms, Konkimalla et al. used pentacyclic triterpenes as a ligand and explored the design of selective inhibitors for the liver and muscle isoforms via molecular docking techniques [16]. Pfizer reported a series of GP inhibitors containing indole structures acting on the new allosteric site. Ingliforib (CP 368296; GPi 296) is one of them and has entered phase II clinical trials [17]. Experiments have shown that ingliforib has IC_50_ values of 52, 352 and 150 nM for the liver, muscle and brain GP, respectively. However, further investigation is needed to elucidate the selective mechanisms of ingliforib for the three GP subtypes.

According to our previous report, when compound **1** acted on the three protein subtypes, a good distinction between the three GP subtypes was obtained compared to other screened compounds [3]. Compound **1** could be considered as a potential lead compound for the design of effective and selective GP inhibitors. Compound **1** mainly consists of two parts: the *N*-(3-(*tert*-butylcarbamoyl)-4-methoxyphenyl) moiety and the indole moiety. In 2000, indole-2-carboxamide analogs were first reported to act on the novel conformational site of HLGPa; they attracted extensive attention due to their unique structural properties [18]. Consequently, the indole unit was introduced into compound **1** for the exploration of specific inhibitors. However, compound **1′**s specific mechanism of action with the three GP subtypes was also not clear. To explore the mechanism of action, in this study, compound **1** and ingliforib were used as the ligands to investigate the binding mode in the three GP isoforms by molecular docking, and the behavior of the complexes was also observed via molecular dynamics simulations. This work is expected to provide guidance for the further structural design and optimization of the highly selective compound.

## 2. Results and Discussion

### 2.1. Binding Model Studies by Molecular Docking

To investigate the associations between compound **1** and various GP subtypes, molecular docking was carried out. Initially, the GP protein structure was acquired from the protein database (refer to Materials and Methods) considering the following criteria: ① human origin; ② conformational resolution ≤ 2.5 Å, minimized to the greatest extent; ③ maximum possible conformational sequence coverage; ④ crystalline pH value as close to the normal physiological range of the human body as feasible. Ensuring that the above four conditions were satisfied to the greatest extent possible, the GP protein structures with PDB IDs of 5IKO (PYGB), 2ZB2 (PYGL) and 1Z8D (PYGM) were chosen for the molecular docking experiments in this study, following appropriate adjustments. Upon completion of molecular docking, the outcomes were examined based on the subsequent aspects.

#### 2.1.1. Spatial Position and Orientation

**Figure 2:**
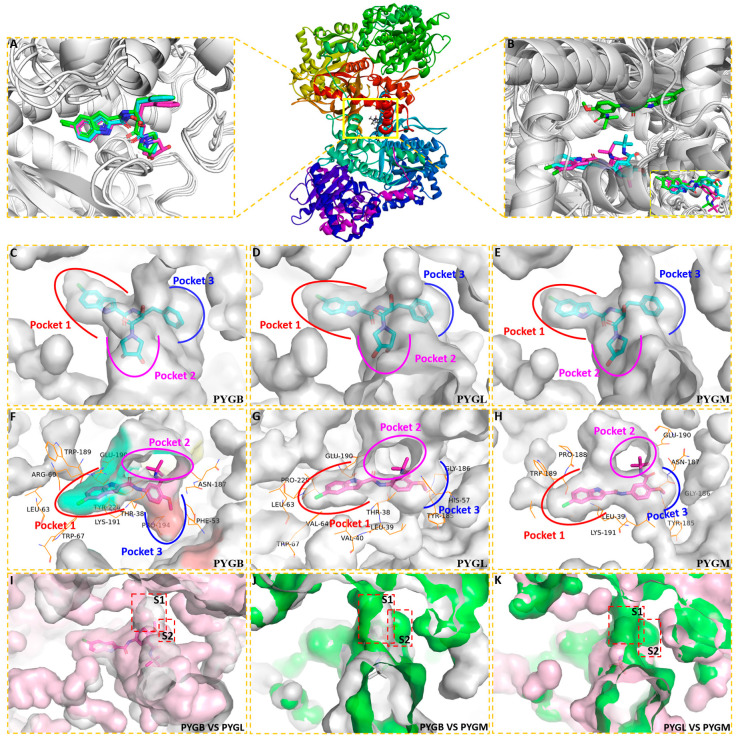
The predicted binding modes of ingliforib and compound **1** with GP by molecular docking (the coloring rules are as follows: PYGB, gray; PYGL, pink; PYGM, green). (**A**) The state of three GP-ingliforib complexes after alignment; (**B**) The state of three GP-compound **1** complexes after alignment; (**C**) Pocket of PYGB-ingliforib; (**D**) Pocket of PYGL-ingliforib; (**E**) Pocket of PYGM-ingliforib; (**F**) Pocket of PYGB-compound **1**; (**G**) Pocket of PYGL-compound **1**; (**H**) Pocket of PYGM-compound **1**; (**I**) PYGB vs. PYGL; (**J**) PYGB vs. PYGM; (**K**) PYGL vs. PYGM.

After docking, the conformation with the lowest binding energy in GP was selected as the probable binding conformation. The spatial position and orientation of the ligand ingliforib and compound **1** in the three GP subtype receptors were observed. As shown in Figure 2A, ingliforib binds within the central cavity consisting of two identical subunits. In the complexes of the three subtypes, the indole, benzyl and dihydroxypyrrolidine moieties of the ligand molecule point towards three different pockets (Figure 2C–E). Specifically, the indole moiety of ingliforib is deeply buried within pocket 1, a closed hydrophobic cavity [19]. The dihydroxypyrrolidine moiety extends towards a wide pocket 2, while the benzyl moiety points towards pocket 3. The ligand molecule adopts a “Y”-shaped conformation at the dimer interface. Comparison of the three subtype complexes using Pymol indicates that their spatial conformations are similar. The differences are mainly observed in dihydroxypyrrolidine, located in pocket 2, which has a different spatial orientation.

For compound **1**, it also binds in a central cavity (Figure 2B) [12,20]. Alignment of the three subtype complexes by Pymol reveals that the spatial location and orientation of the ligand in PYGL are different from those of the other two subtypes. However, after rotation, PYGL can be found to be in essentially the same position as the other two ligands. This leads to the inference that the ligand in PYGL may have entered another similar pocket in the dimer cavity with reverse symmetry. This may be reasonable as the site is located at the interface of a dimer composed of two identical subunits. Previous X-ray crystallography studies have demonstrated that two indole molecules of the GP inhibitor CP526423 bind identically within the solvent cavity at the dimer interface, within 6Å of each other [21]. In the three subtype complexes, the conformation of compound **1** in the pocket shows an approximate “Y” shape too (Figure 2F–H), i.e., the indole, tert-butylamide group and methoxy point to three different pockets. The indole portion of compound **1** is positioned deep within pocket 1. The tert-butyl amide fragment points to a wide pocket 2. The methoxy fragment is in pocket 3. Comparison of the GP subtype complexes shows that the methoxy fragment of pocket 3 is not identical, with the ligand molecule in PYGB occupying the S1 region and the ligands in PYGL and PYGM occupying mainly the S2 region (Figure 2I–K), which is also different from ingliforib. This may be due to the spatial or conformational constraints of the three GP isoforms. In addition, the ligand molecule in PYGB has room for further growth in pocket 3. In short, the ligand molecules exhibit a “Y”-shaped conformation at the dimer interface and there is a slight difference in pocket 3 occupied by methoxy. The difference in ligand conformation aroused our interest in their binding modes. Therefore, the binding mode was analyzed afterwards.

#### 2.1.2. Binding Mode

**Figure 3:**
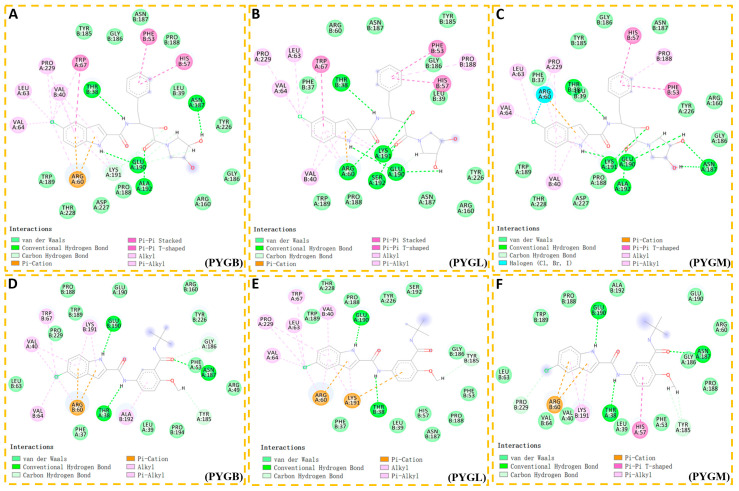
Binding modes of inhibitor ingliforib and compound **1** docking with three GP subtypes. (**A**) PYGB-ingliforib; (**B**) PYGL-ingliforib; (**C**) PYGM-ingliforib; (**D**) PYGB-compound **1**; (**E**) PYGL-compound **1**; (**F**) PYGM-compound **1**.

Next, to illustrate the interaction mechanism, the binding modes of the ingliforib and compound **1** to the three isoforms were analyzed. The molecular docking results showed that the docking scores of ingliforib with PYGB, PYGL and PYGM were −10.782, −10.854 and −9.872 kcal/mol, respectively. Based on the molecular docking figures, although the binding modes of the three complexes were similar, some differences were also observed. In PYGB, the indole NH of ingliforib formed a hydrogen bond with Glu190, the indole formed a cation–π interaction with Lys191, the amide NH of the linker formed a hydrogen bond with Thr38′, the phenyl ring formed a π–π interaction with His57′, the carbonyl oxygen of the side chain amide formed a hydrogen bond with Ala192 and another hydroxyl group formed a hydrogen bond with Asn187 (Figure 3A). The rich receptor–ligand interactions endowed ingliforib with good inhibitory activity against PYGB. In PYGL, ingliforib also had abundant receptor–ligand interactions and additionally formed a hydrogen bond with Arg60, resulting in the best inhibitory activity against PYGL (Figure 3B). However, in PYGM, ingliforib exhibited relatively poor inhibitory activity due to the lack of a cation–π interaction with Lys191 or a hydrogen bond interaction with Arg60, although it had similar receptor–ligand interactions as in PYGB (Figure 3C).

The docking scores of compound **1** with PYGB, PYGL and PYGM were −8.329, −7.244 and −8.266 kcal/mol, respectively. In PYGB, compound **1** formed hydrogen bonds with Glu190 and Thr38′, and a carbonyl oxygen of its side chain amide formed a hydrogen bond with Asn187′. Additionally, Lys191 formed a cation–π interaction with the indole (Figure 3D). In PYGL, compound **1** lost the hydrogen bond with Asn187′ and the carbon hydrogen bond interaction with Gly186′ (Figure 3E). In PYGM, except for the loss of a carbon–hydrogen bond interaction with the isobutylamide group, all other hydrogen bonds were consistent with PYGB. Furthermore, the indole formed a cation–π interaction with Arg60, but its alkyl and π–alkyl interactions with some amino acid residues were weakened, and His57′ formed a π–π T-shaped interaction with the phenyl ring (Figure 3F). This may be an important reason for the activity differences observed among the ligands in the three GP subtypes [3].

In comparing the complex of compound **1** with that of ingliforib, it was found that for the same subtype, the differences in their structures resulted in certain variations in the ways in which they interacted with amino acid residues. For example, in PYGL, the dihydroxy-pyrrolidine group of ingliforib in pocket 2 formed hydrogen bonds with Glu190′ and Ser192′, whereas this was not observed in compound **1**. Additionally, the ways in which they interacted with the same amino acid residue could also be different. For instance, in PYGB, the tail benzene ring of compound **1** formed a π–alkyl interaction with ALA192. Such differences might explain why the two compounds exhibited distinct selectivity towards the three GP subtypes. In summary, the results suggested that the conformation of ligand compound **1** was stabilized in the three GP isoforms via hydrophobic interactions, hydrogen bonding and π–cation stacking, which is consistent with previous findings [16,20]. The indole ring as well as the two amide parts were considered to be the key parts of the activity.

### 2.2. Molecular Dynamics Simulations

Based on the molecular docking results of ingliforib and compound **1** with the different subtypes of GP, molecular dynamics simulation was employed to explore the stability of their interactions and the key amino acid residues involved. The simulation was set for 50 ns with the initial structure as a reference, and the RMSD values were calculated to reflect the stability of the receptor–ligand complex conformation during the simulation. The hydrogen bond occupancy rate was calculated based on the stable trajectory to assess the stability of the hydrogen bond interactions. Additionally, the binding free energy was calculated, and energy decomposition was performed to determine which amino acid residues contributed significantly to the binding of compound **1** and ingliforib.

**Figure 4:**
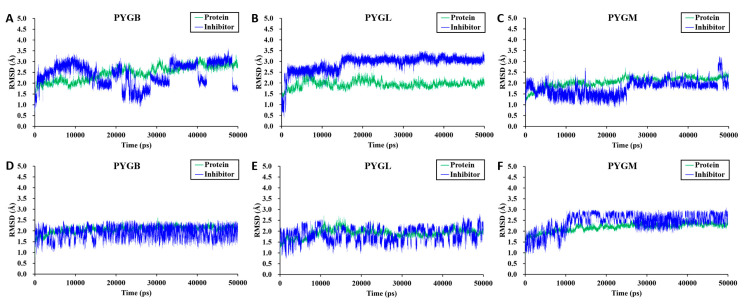
Time courses of RMSD for complexes. (**A**) RMSD of PYGB-ingliforib; (**B**) RMSD of PYGL-ingliforib; (**C**) RMSD of PYGM-ingliforib; (**D**) RMSD of PYGB-compound **1**; (**E**) RMSD of PYGL-compound **1**; (**F**) RMSD of PYGM-compound **1**.

In Figure 4A, the PYGB–ingliforib complex exhibits fluctuations during the 50 ns simulation. Beginning at 30 ns, ingliforib’s RMSD value fluctuates around 3.0 Å, with periodic jumps within the range of 2.0–2.5 Å. PYGB’s RMSD value remains stable around 2.5 Å. The PYGL–ingliforib complex remains stable throughout the simulation (Figure 4B), with the RMSD value of PYGL uniformly fluctuating between 1.5 and 2.0 Å. Prior to 15 ns, ingliforib’s RMSD value for the PYGM–ingliforib complex fluctuates uniformly around 2.5 Å, while, after 15 ns, it fluctuates uniformly around 3.0 Å. During the simulation, the RMSD of PYGM fluctuates around 2.0 Å before 25 ns and shows an increasing trend, while the RMSD of ingliforib remains uniformly fluctuating around 1.5 Å. After 25 ns, both PYGM and ingliforib maintain uniform fluctuations around 2.0 Å and 1.5–2.0 Å, respectively, with occasional jumps in ingliforib’s RMSD value (Figure 4C).

For compound **1**, we can observe that the PYGB–compound **1** complex remained relatively stable during the 50 ns simulation. The RMSD value of the protein PYGB fluctuated within approximately 2.0 Å, while compound **1** mainly fluctuated uniformly in the range of 1.25–2.25 Å (Figure 4D). Similarly, the PYGL–compound **1** complex also remained stable (Figure 4E). After 10 ns, the protein predominantly fluctuated around 2.0 Å, while compound **1** fluctuated between 1.25 and 2.25 Å. For the PYGM–compound **1** complex (Figure 4F), equilibrium was reached after 10 ns, and the protein fluctuated mainly between 2.0 and 2.5 Å, while compound **1** fluctuated between 2.0 and 3.0 Å. Overall, the fluctuation range of the compound 1–GP complex was narrower than that of the ingliforib–protein complex.

Considering the memory required to calculate hydrogen bond occupancy rates, the stable molecular dynamics simulation trajectories between 45 and 50 ns were selected to calculate the hydrogen bond occupancy rates. Comparing the hydrogen bond occupancy rates between the three receptor–ligand complexes (Table 1), it can be observed that both PYGB–compound **1** and PYGL–compound **1** maintained a stable binding conformation with the molecular docking and formed a stable hydrogen bond with Glu190. Additionally, in PYGL–compound **1**, the compound formed stable hydrogen bond interactions with Thr38′ and His57′. However, the hydrogen bond between the compound and Glu190 was not maintained in PYGM–compound **1**, but compound **1** formed a stable hydrogen bond with His57, with an occupancy rate of 99.85%, indicating that the conformation of the compound may have undergone some flipping. In PYGB–compound **1** and PYGM–compound **1**, the intramolecular hydrogen bonds of compound **1** were relatively stable, with hydrogen bond occupancy rates of 59.03% and 91.72%, respectively, suggesting that the tert-butyl carbamoyl amide may have undergone some flipping. Although there were also intramolecular hydrogen bonds in PYGL–compound **1**, the occupancy rate was only 11.74%. These trajectories reflected that compound **1** could obtain relatively stable binding conformations after binding to the three subtypes of GP.

The stable molecular dynamics simulation trajectories between 45 and 50 ns of the ingliforib–protein complexes were selected to calculate the hydrogen bond occupancy and binding free energy. The results of the hydrogen bond occupancy are presented in Table 1. In PYGB–ingliforib, stable hydrogen bonds were formed between Glu190 and Lys191 and ingliforib, with hydrogen bond occupancy of 96.86% for Glu190. Ingliforib also showed some intramolecular hydrogen bonding interactions. In PYGL–ingliforib, ingliforib formed stable hydrogen bonds with Thr38′, Glu190 and Ser192, with high hydrogen bond occupancies. The hydrogen bond occupancy with Ser192 reached 76.07%. In PYGM–ingliforib, ingliforib primarily formed stable hydrogen bonds with Glu190, Thr38′ and Lys191, along with some intramolecular hydrogen bonds. Comparing the three systems, it was found that the stable hydrogen bond interaction with Glu190 was present in all three systems. The hydrogen bond interaction with Lys191 was present in PYGB and PYGM, but not in PYGL. The hydrogen bond interaction with Thr38′ was absent in PYGB, suggesting that this hydrogen bond may not be necessary for the inhibitory activity of the compound against PYGB. The stable hydrogen bond interaction with Ser192 was only present in PYGL, indicating that it may enhance the selectivity of the compound for PYGL.

### 2.3. Calculation of Binding Free Energy Based on MM-PBSA

**Figure 5:**
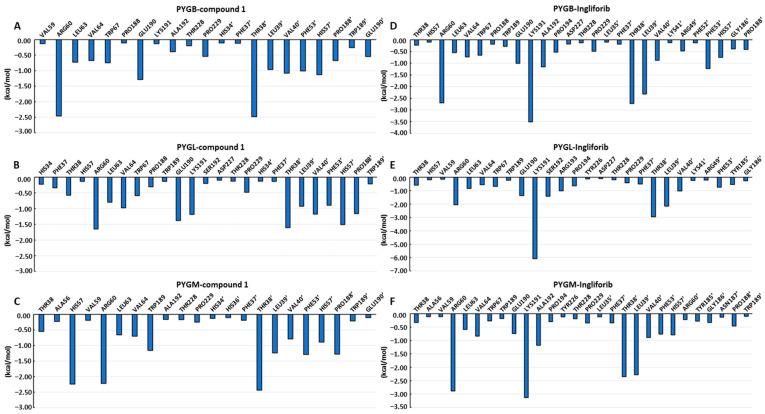
Per-residue energy decomposition of binding free energy calculated by MM-PBSA method.

On the basis of the MD simulation, the MM-PBSA method was employed for the calculation of the binding free energies of compound **1** with the three subtypes of GP (Table 2), which were found to be −38.26 ± 1.61, −37.61 ± 2.08 and −35.10 ± 2.57 kcal/mol for compound **1** with PYGB, PYGM and PYGL, respectively. This indicates that compound **1** has the strongest binding affinity with PYGB, followed by PYGM and then PYGL (IC_50_ of PYGB, PYGL and PYGM, which are 0.11 ± 0.01 µM, 0.35 ± 0.02 µM and 0.93 ± 0.01 µM, respectively) [3]. The strong correlation observed between the computed interaction free energies and the inhibitory activity obtained experimentally indicates that the binding conformations of the inhibitors are reasonable. Then, the binding free energy was further decomposed to identify the amino acid residues that contribute significantly to compound **1′**s binding between 45 and 50 ns, focusing on residues whose contribution values are greater than −0.01 kcal/mol, with values exceeding −1.00 kcal/mol considered important. In the PYGB–compound **1** complex (Figure 5A), Arg60, Glu190, Thr38′, Val40′, Phe53′ and His57′ were found to make a significant contribution to compound **1′**s binding with PYGB. In the PYGL–compound **1** complex (Figure 5B), Arg60, Val64, Glu190, Lys191, Thr38′, Val40′, His57′ and Pro188′ were identified as important residues for compound **1′**s binding with PYGL. In the PYGM–compound **1** complex (Figure 5C), His57, Arg60, Trp189, Thr38′, Leu39′, Phe53′ and Pro188′ were found to have significant contributions to compound **1′**s binding with PYGM. These results indicate that amino acid residues on both monomers of the protein dimer contribute significantly to compound **1′**s binding. In comparison, Pro188′ had a relatively small contribution value in the PYGB–compound **1** system, but it was an important residue in both the PYGL–compound **1** and PYGM–compound **1** systems. Additionally, Glu190 played an important role in the binding of compound **1** to both PYGB and PYGL, while it was not an important residue in the PYGM–compound **1** system, despite the substantial contribution of Trp189. Therefore, we concluded that maintaining interactions with Arg60, Glu190 and Thr38′, as well as interacting with Leu39′, Val40′, Phe53′ and His57′ on the other monomer, while reducing interactions with Trp189 and Pro188′, can lead to improved selectivity for PYGB.

As demonstrated in Table 2, the strongest binding was between ingliforib and PYGL, followed by PYGB, and the weakest was with PYGM. The energy decomposition analysis revealed the importance of certain amino acid residues in ingliforib binding. For the PYGB–ingliforib complex (Figure 5D), Arg60, Glu190, Lys191, Ala192, Thr38′, Leu39′ and Phe53′ were found to make significant contributions to the binding. For the PYGL–ingliforib complex (Figure 5E), Arg60, Glu190, Lys191, Ser192, Arg193, Thr38′, Leu39′ and Val40′ were identified as key contributors. In the PYGM–ingliforib complex (Figure 5F), Arg60, Lys191, Ala192, Thr38′ and Leu39′ were crucial for the binding. It can be seen that the amino acid residues discovered through energy decomposition make significant contributions to the binding of the compound. Comparing the three proteins, maintaining mutual interactions between Arg60, Glu190 and Lys191, while interacting with Thr38′ and Leu39′ of the other half of the protein, is necessary for the inhibitory activity of the compound against all three proteins. Moreover, hydrogen bonding with Ser192 can increase the selectivity of the compound for PYGL, since Ala192 is present in both PYGB and PYGM. Lys191 also plays a critical role in the structure of the compound–PYGL complex, with its contribution being twice that in PYGB and PYGM, suggesting that enhancing the interaction between Lys191 and the compound would improve its selectivity against PYGL. In addition, Arg193 is also important for the structure of the complex, while its contribution in PYGB and PYGM is not significant; thus, increasing the interaction between the compound and Arg193 would also be advantageous for its selectivity against PYGL.

### 2.4. Pharmacophore Modeling

To provide better theoretical guidance for the design of new inhibitors, here, we provide some references for the design of new compounds. Based on the experimental results, maintaining the interactions between Arg60, Glu190 and Lys191, while forming interactions with Thr38′ and Leu39′ of the other half of the protein monomer, are some key factors required for the inhibitory activity of the compound against GP. For PYGL, given that the amino acid residue 192 is Ala in both PYGB and PYGM but Ser in PYGL, the formation of a strong interaction with Ser192 would facilitate the selectivity of the compound for PYGL. Conversely, strengthening the interactions between the compound and Ala192 is also beneficial to improve its selectivity against PYGB and/or PYGM. Therefore, introducing corresponding functional groups or structures into the compound to enhance the interactions with Ser192 could be considered. In PYGB, a strategy may be to enhance the interaction of the compound with the amino acid residues around the S1 region that make up pocket 3 through structural modifications.

**Figure 6:**
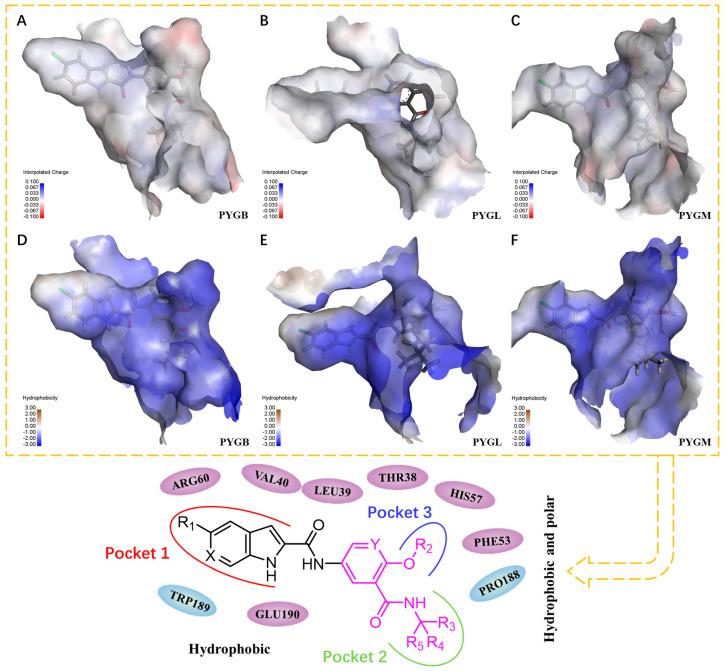
Compound **1** in three subtypes of GP is displayed by mesh and surface and pharmacophore modeling. (**A**) Interpolated charge of PYGB; (**B**) Interpolated charge of PYGL; (**C**) Interpolated charge of PYGM; (**D**) Hydrophobicity of PYGB; (**E**) Hydrophobicity of PYGL; (**F**) Hydrophobicity of PYGM.

In terms of compound structure, for the indole portion of pocket 1, the indole unit is constrained in a relatively narrow sub-pocket (pocket 1). Although the scope for the growth of the indole unit is limited, it may be a better strategy to try to substitute it according to the bioelectronic isomer rule. For the methoxy fragment in pocket 3, introducing electron-withdrawing groups to enhance its interaction may be effective due to the electronegative region at the bottom of the pocket (Figure 6A), which may not be valid for PYGL and PYGM (Figure 6B,C). As shown in Figure 6D–F, attempting to introduce more hydrophilic groups while maintaining the original hydrogen bonds in the ligand portion of pocket 2 may be effective in further improving the overall activity (forming surface colors ranging from hydrophilic blue to hydrophobic brown based on the hydrophobicity of the receptor residues). The above findings may offer some contribution to the design of GP inhibitors specifically against GP isoenzymes.

In summary, in the GP–compound **1** or GP–ingliforib complexes, there was some variability in the amino acids involved and their contributions. Therefore, modifications should be made to the compound according to specific situations for different protein systems, to enhance or weaken its interactions with certain amino acid residues. Meanwhile, the spatial structure of the compound should be further optimized to better fit the active site of the target protein and increase its non-covalent interactions with the target protein.

## 3. Materials and Methods

### 3.1. Molecular Docking

Firstly, molecular docking was performed using the Glide module of Schrödinger2021. The PDB files 5IKO [13,22], 2ZB2 [23,24] and 1Z8D [25,26] were used for PYGB, PYGL and PYGM, respectively. The dimer was directly downloaded from the UniProt database. For Tetramer, delete the redundant monomer to obtain the dimer. Although only 2ZB2 has a complex crystal structure of PYGL with a small molecule, information about the binding sites of small molecules in PYGB and PYGM can be obtained by superimposing them based on the sequence homology. According to the crystal structures, small molecules bind to the middle site of the protein dimer. Consequently, a grid file of the binding site was generated using Glide Grid Generation. The Ligpre module was utilized to process small molecules with the OPLS4 force field setting, hydrogenation, ionization and energy optimization. Finally, molecular docking was conducted using the Glide XP (extra precision) mode. The docking was semi-flexible, i.e., the ligand conformation was flexible while the pocket was fixed. The grid file for the binding site was defined by a box centered on the crystal ligand with a similar size. No constraints were included during grid generation. The Glide XP docking score was used to rank the ligand positions. Protonation of the residues was automatically determined under the neutral condition using the Protein Preparation Wizard workflow. Results were visualized using Pymol and Discovery Studio 4.5 Visualizer.

### 3.2. Molecular Dynamics Simulation

The molecular dynamics simulation utilized the GROMACS 2019.6 package [27], with the CHARMM 36 force field [28,29,30,31] the TIP4P water model and suitable ion parameters [32,33] as well as ligand force field parameters generated with the SwissParam webserver [34]. The protein–ligand complex was first energy-minimized in a vacuum, followed by immersion in a dodecahedral periodic box with three-dimensional boundary conditions, at a minimum distance of 12 Å from the box frame. To simulate a physiological environment, the simulation box was filled with TIP4P model water molecules, and sodium and chloride ions were added to balance the system charge at an ion concentration of 150 mM. After solvation, the protein’s energy was minimized again with positional restraints applied to its backbone atoms, while allowing the solvent to freely diffuse. Equilibration of the system continued with a 1 ns NVT run at 300 K (using a heat bath based on the v-rescale algorithm with EnerPres correction), followed by a 1 ns NPT run at 1 atm (using the Parrinello Rahman the pressure controller), both still with positional restraints. NPT collection runs with no positional restraints were 50 ns long. The LinCS method constrained all bonds. Particle Mesh Ewald (PME) was used for long-range electrostatic interactions, with a cutoff value of 10 Å. The program provided with GROMACS 2019.6 was used to determine the root mean square deviation (RMSD). The complexes for each GP subtype in complex with the compounds obtained by molecular docking were used as the initial conformations for molecular dynamic simulations and also used as the reference structures to calculate the RMSD values. The results were visualized using Pymol and Discovery Studio 4.5 Visualizer.

### 3.3. Calculation of Binding Free Energy Based on MM-PBSA Method

The estimation of the interaction free energy was performed using the molecular mechanics Poisson Boltzmann surface area (MM-PBSA) approach. The binding free energy was computed through the MM-PBSA method with g_mmpbsa [35,36]. The system’s enthalpy was evaluated using the molecular mechanics (mm) approach, and the contributions of the polar and non-polar components of the solvent effect to the free energy were assessed by resolving the Poisson Boltzmann (PB) equation and computing the molecular surface area (SA). The vacuum potential energy, polar solvation energy and non-polar solvation energy were ascertained using g_mmpbsa computation. The Python script included in the g_mmpbsa package was employed to determine the mean binding energy and standard deviation.

## 4. Conclusions

In this study, the interaction mechanisms between ingliforib and compound **1** and PYGB, PYGL and PYGM were elucidated through molecular docking and molecular dynamics simulations. Observations revealed that certain dissimilarities existed in the three protein isoforms with regard to their spatial conformations, and corresponding differences were also evident in the binding modes of the ligands. These conformations were stabilized by interactions such as hydrophobic interactions, hydrogen bonds and π–cation stacking. The rich receptor–ligand interactions endowed ingliforib and compound **1** with good specific inhibitory activity against PYGL and PYGB, respectively. Subsequently, these results were validated through molecular dynamics simulations. The RMSD values of the protein–ligand complexes during 50 ns of molecular dynamics simulation remained stable within a specific range. The hydrogen bond occupancy and binding free energy were further calculated using the MM-PBSA method, and some key amino acid residues in each system that significantly contributed to ligand binding were identified. These findings provide a theoretical basis for the optimization or design of new GP inhibitors to achieve better inhibitory activity against specific target proteins.

## Figures and Tables

**Table 1:** Hydrogen bond occupancy rates of compound **1** or ingliforib with PYGB, PYGL and PYGM based on stable trajectories.

Compound	Acceptor	Donor	Occupancy (%)	Distance (Å)	Angle (°)
PYGB–compound **1**	Glu190@O	MOL@H1N1	97.16	2.938	148.2
	Thr38′@O	MOL@H3N2	3.82	2.949	148.5
	MOL@O2	His57′@HE2NE2	3.31	3.088	147.5
PYGL–compound **1**	Glu190@O	MOL@H4N1	99.74	2.893	157.6
	Thr38′@O	MOL@H6N2	94.15	2.955	146.6
	MOL@O2	His57′@HE2NE2	75.24	3.074	148.9
	MOL@O3	Arg60′@HH12NH1	20.00	2.858	158.0
	MOL@O3	Arg60′@HH22NH2	9.31	3.173	137.8
	MOL@O3	His57′@HE2NE2	8.16	3.183	131.5
PYGM–compound **1**	His57@ND1	MOL@H1N1	99.85	2.936	155.3
	MOL@O2	His57′@HE2NE2	23.01	3.019	141.0
	MOL@O3	Lys191@HZ2NZ	22.91	2.818	149.7
	Thr38′@O	MOL@H3N2	19.77	3.149	135.0
	MOL@O3	Lys191@HZ1NZ	13.89	2.811	148.0
	MOL@O3	Lys191@HZ3NZ	10.98	2.837	149.6
PYGB–ingliforib	Glu190@O	MOL@H4N1	96.86	2.929	149.8
	MOL@O5	Lys191@HZ3NZ	37.16	2.804	153.2
	MOL@O5	Lys191@HZ1NZ	29.79	2.792	154.7
	MOL@O5	Lys191@HZ2NZ	25.25	2.791	154.4
PYGL–ingliforib	Thr38′@O	MOL@H21O2	99.43	2.761	159.3
	Glu190@O	MOL@H4N1	99.03	2.857	153.7
	Thr38′@O	MOL@H6N2	93.73	3.129	139.1
	Ser192@O	MOL@H22O3	76.07	2.679	160.3
PYGM–ingliforib	Glu190@O	MOL@H4N1	99.91	2.863	154.5
	Thr38′@O	MOL@H21O2	97.39	2.835	151.9
	Thr38′@OG1	MOL@H6N2	67.59	3.151	149.5
	Thr38′@O	MOL@H6N2	58.24	3.090	136.0
	MOL@O5	Lys191@HZ1NZ	32.18	2.816	149.7
	MOL@O5	Lys191@HZ3NZ	30.03	2.813	149.5
	MOL@O5	Lys191@HZ2NZ	24.19	2.807	150.5

The atomic numbers of compound **1** and ingliforib are shown in Supplementary Materials Figures S7 and S8.

**Table 2:** Binding energy between ligands and GP through MM-PBSA estimation.

**Energy Component (kcal·mol^−1^)**	**Compound 1**		
	**PYGB**	**PYGL**	**PYGM**
ELE	−31.72 ± 4.15	−21.51 ± 4.97	−32.07 ± 4.36
VDW	−46.75 ± 2.05	−47.85 ± 3.04	−47.56 ± 2.1
GAS	−78.47 ± 3.34	−69.36 ± 2.78	−79.63 ± 3.68
GBSUR	−5.86 ± 0.11	−6.26 ± 0.19	−6.4 ± 0.14
GB	46.08 ± 3.15	40.53 ± 2.77	48.42 ± 2.89
GBSOL	40.21 ± 3.19	34.27 ± 2.89	42.02 ± 2.82
GBELE	14.35 ± 1.83	19.02 ± 3.22	16.35 ± 2.59
GBTOT	−38.26 ± 1.61	−35.10 ± 2.57	−37.61 ± 2.08
**Energy Component (kcal·mol^−1^)**	**Ingliforib**		
	**PYGB**	**PYGL**	**PYGM**
ELE	−54.05 ± 6	−50 ± 5.64	−48.15 ± 2.56
VDW	−50.9 ± 1.14	−51.97 ± 4.01	−46.4 ± 5.64
GAS	−104.95 ± 6.74	−101.97 ± 3.51	−94.55 ± 7.39
GBSUR	−6.45 ± 0.25	−6.98 ± 0.1	−6.52 ± 0.33
GB	70.65 ± 8.84	66.72 ± 4.53	62.45 ± 3.27
GBSOL	64.2 ± 8.69	59.74 ± 4.45	55.94 ± 2.94
GBELE	16.6 ± 2.91	16.72 ± 2.18	14.3 ± 1.62
GBTOT	−40.75 ± 1.96	−42.23 ± 1.91	−38.62 ± 4.52

ELE = electrostatic energy as calculated by the MM force field. VDW = van der Waals contribution from MM. GAS = total gas phase energy. GBSUR = non-polar contribution to the solvation free energy calculated by an empirical model. GB = the electrostatic contribution to the solvation free energy calculated by PB or GB, respectively. GBSOL = sum of non-polar and polar contributions to solvation. GBELE = sum of the electrostatic solvation free energy and MM electrostatic energy. GBTOT = final estimated binding free energy calculated from the terms above.

## Data Availability

The data presented in this study are available on request from the corresponding author.

## Supplementary Materials

The following supporting information can be downloaded at: https://www.mdpi.com/article/10.3390/molecules28134909/s1, Figure S1: Predicted binding mode of molecular docking between compound **1** and PYGB; Figure S2: Predicted binding mode of molecular docking between compound **1** and PYGL; Figure S3: Predicted binding mode of molecular docking between compound **1** and PYGM; Figure S4: Predicted binding mode of molecular docking between Ingliforib and PYGB; Figure S5: Predicted binding mode of molecular docking between Ingliforib and PYGL; Figure S6: Predicted binding mode of molecular docking between Ingliforib and PYGM; Figure S7: Atomic number of compound **1**; Figure S8: Atomic number of ingliforib.

## References

[1] Qurtam A.A., Mechchate H., Es-Safi I., Al-Zharani M., Nasr F.A., Noman O.M., Aleissa M., Imtara H., Aleissa A.M., Bouhrim M. (2021). Citrus Flavanone Narirutin, In Vitro and In Silico Mechanistic Antidiabetic Potential. Pharmaceutics.

[2] Huang Y., Li S., Wang Y., Yan Z., Guo Y., Zhang L. (2023). A Novel 5-Chloro-N-phenyl-1H-indole-2-carboxamide Derivative as Brain-Type Glycogen Phosphorylase Inhibitor: Validation of Target PYGB. Molecules.

[3] Yan Z., Li S., Wang Y., Li J., Ma C., Guo Y., Zhang L. (2022). Discovery of novel heterocyclic derivatives as potential glycogen phosphorylase inhibitors with a cardioprotective effect. Bioorg. Chem..

[4] Curtis M., Kenny H.A., Ashcroft B., Mukherjee A., Johnson A., Zhang Y., Helou Y., Batlle R., Liu X., Gutierrez N. (2019). Fibroblasts Mobilize Tumor Cell Glycogen to Promote Proliferation and Metastasis. Cell Metab..

[5] Cai Y., Guo H., Fan Z., Zhang X., Wu D., Tang W., Gu T., Wang S., Yin A., Tao L. (2020). Glycogenolysis Is Crucial for Astrocytic Glycogen Accumulation and Brain Damage after Reperfusion in Ischemic Stroke. iScience.

[6] Agius L. (2007). New hepatic targets for glycaemic control in diabetes. Best. Pract. Res. Clin. Endocrinol. Metab..

[7] Guan T., Qian Y., Tang X., Huang M., Huang L., Li Y., Sun H. (2011). Maslinic acid, a natural inhibitor of glycogen phosphorylase, reduces cerebral ischemic injury in hyperglycemic rats by GLT-1 up-regulation. J. Neurosci. Res..

[8] Migocka-Patrzałek M., Elias M. (2021). Muscle Glycogen Phosphorylase and Its Functional Partners in Health and Disease. Cells.

[9] Chrysina E.D. (2010). The prototype of glycogen phosphorylase. Mini-Rev. Med. Chem..

[10] Aiston S., Hampson L., Gómez-Foix A.M., Guinovart J.J., Agius L. (2001). Hepatic Glycogen Synthesis Is Highly Sensitive to Phosphorylase Activity. J. Biol. Chem..

[11] Fletterick R.J., Sygusch J., Semple H., Madsen N.B. (1976). Structure of glycogen phosphorylase a at 3.0 A resolution and its ligand binding sites at 6 A. J. Biol. Chem..

[12] Oikonomakos N.G., Skamnaki V.T., E Tsitsanou K., Gavalas N.G., Johnson L.N. (2000). A new allosteric site in glycogen phosphorylase b as a target for drug interactions. Structure.

[13] Mathieu C., de la Sierra-Gallay I.L., Duval R., Xu X., Cocaign A., Léger T., Woffendin G., Camadro J.-M., Etchebest C., Haouz A. (2016). Insights into Brain Glycogen Metabolism. J. Biol. Chem..

[14] Mathieu C., Dupret J.-M., Rodrigues-Lima F. (2016). The structure of brain glycogen phosphorylase-from allosteric regulation mechanisms to clinical perspectives. FEBS J..

[15] Freeman S., Bartlett J.B., Convey G., Hardern I., Teague J.L., Loxham S.J.G., Allen J.M., Poucher S.M., Charles A.D. (2006). Sensitivity of glycogen phosphorylase isoforms to indole site inhibitors is markedly dependent on the activation state of the enzyme. Br. J. Pharmacol..

[16] Konkimalla V. (2017). An Improved Comparative Docking Approach for Developing Specific Glycogen Phosphorylase Inhibitors Using Pentacyclic Triterpenes. Curr. Top. Med. Chem..

[17] Tracey W.R., Treadway J.L., Magee W.P., Sutt J.C., McPherson R.K., Levy C.B., Wilder D.E., Yu L.J., Chen Y., Shanker R.M. (2004). Cardioprotective effects of ingliforib, a novel glycogen phosphorylase inhibitor. Am. J. Physiol. Heart Circ. Physiol..

[18] Chehardoli G., Bahmani A. (2020). Synthetic strategies, SAR studies, and computer modeling of indole 2 and 3-carboxamides as the strong enzyme inhibitors: A review. Mol. Divers..

[19] Henke B.R., Sparks S.M. (2006). Glycogen phosphorylase inhibitors. Mini Rev. Med. Chem..

[20] Rath V.L., Ammirati M., Danley D.E., Ekstrom J.L., Gibbs E.M., Hynes T.R., Mathiowetz A.M., McPherson R.K., Olson T.V., Treadway J.L. (2000). Human liver glycogen phosphorylase inhibitors bind at a new allosteric site. Chem. Biol..

[21] Zhang L., Chen X., Liu J., Zhu Q., Leng Y., Luo X., Jiang H., Liu H. (2012). Discovery of novel dual-action antidiabetic agents that inhibit glycogen phosphorylase and activate glucokinase. Eur. J. Med. Chem..

[22] PYGB—Glycogen Phosphorylase, Brain Form—Homo Sapiens (Human) UnitProtKB|Uniprot. https://www.uniprot.org/uniprot/P11216.

[23] Onda K., Suzuki T., Shiraki R., Yonetoku Y., Negoro K., Momose K., Katayama N., Orita M., Yamaguchi T., Ohta M. (2008). Synthesis of 5-chloro-N-aryl-1H-indole-2-carboxamide derivatives as inhibitors of human liver glycogen phosphorylase a. Bioorg. Med. Chem..

[24] PYGL—Glycogen Phosphorylase, Liver Form—Homo Sapiens (Human) UnitProtKB|Uniprot. https://www.uniprot.org/uniprot/P06737.

[25] Lukacs C.M., Oikonomakos N.G., Crowther R.L., Hong L.N., Kammlott R.U., Levin W., Li S., Liu C.M., Lucas-McGady D., Pietranico S. (2006). The crystal structure of human muscle glycogen phosphorylase a with bound glucose and AMP: An intermediate conformation with T-state and R-state features. Proteins.

[26] PYGM—Glycogen Phosphorylase, Muscle Form—Homo Sapiens (Human)|UnitProtKB|Uniprot. https://www.uniprot.org/uniprot/P11217.

[27] Hess B., Kutzner C., Van Der Spoel D., Lindahl E. (2008). GROMACS 4: Algorithms for Highly Efficient, Load-Balanced, and Scalable Molecular Simulation. J. Chem. Theory Comput..

[28] MacKerell A.D., Bashford D., Bellott M., Dunbrack R.L., Evanseck J.D., Field M.J., Fischer S., Gao J., Guo H., Ha S. (1998). All-atom empirical potential for molecular modeling and dynamics studies of proteins. J. Phys. Chem. B.

[29] MacKerell A.D., Feig M., Brooks C.L. (2004). Improved treatment of the protein backbone in empirical force fields. J. Am. Chem. Soc..

[30] Best R.B., Zhu X., Shim J., Lopes P.E., Mittal J., Feig M., Mackerell A.D. (2012). Optimization of the additive CHARMM all-atom protein force field targeting improved sampling of the backbone phi, psi and side-chain chi(1) and chi(2) dihedral angles. J. Chem. Theory Comput..

[31] Huang J., Rauscher S., Nawrocki G., Ran T., Feig M., de Groot B.L., Grubmuller H., MacKerell A.D. (2017). CHARMM36m: An improved force field for folded and intrinsically disordered proteins. Nat. Methods.

[32] Jorgensen W.L., Chandrasekhar J., Madura J.D., Impey R.W., Klein M.L. (1983). Comparison of simple potential functions for simulating liquid water. J. Chem. Phys..

[33] Beglov D., Roux B. (1994). Finite representation of an infinite bulk system: Solvent boundary potential for computer simulations. J. Chem. Phys..

[34] Zoete V., Cuendet M.A., Grosdidier A., Michielin O. (2011). SwissParam: A fast force field generation tool for small organic molecules. J. Comput. Chem..

[35] Baker N.A., Sept D., Joseph S., Holst M.J., McCammon J.A. (2001). Electrostatics of nanosystems: Application to microtubules and the ribosome. Proc. Natl. Acad. Sci. USA.

[36] Kumari R., Kumar R., Lynn A. (2014). g_mmpbsa—A GROMACS tool for high-throughput MM-PBSA calculations. J. Chem. Inf. Model..

