# Supplementary material for: Computational Insights into Novel Inhibitor N-(3-(tert-Butylcarbamoyl)-4-methoxyphenyl)-indole and Ingliforib Specific against GP Isoenzyme Dimers Interaction Mechanism
Source: Molecules. 2023 Jun 22;28(13):4909. doi: 10.3390/molecules28134909 (PMC10343391; doi:10.3390/molecules28134909)
Supplement: Supplementary file 1 [file molecules-28-04909-s001.zip › molecules-2405092 -Supporting information.pdf]

Supporting Information

Computational insights into novel inhibitor N-(3-(tert-butylcarbamoyl)-4-methoxyphenyl)-indole and ingliforib specific against GP isoenzyme dimers interaction mechanism

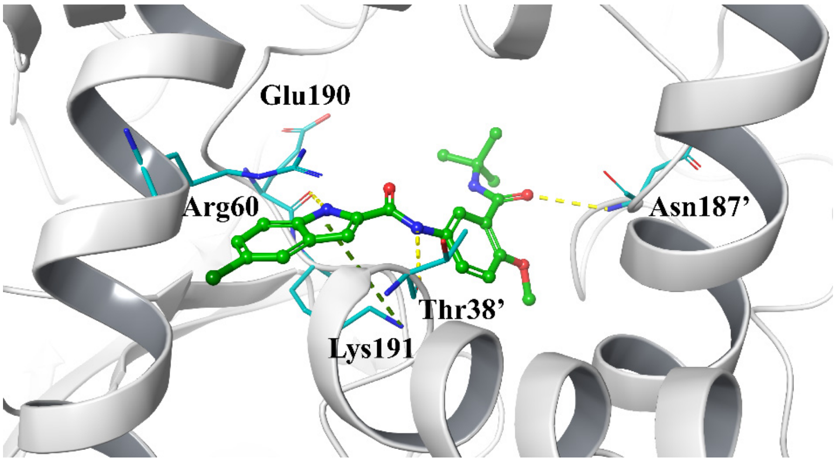

Figure S1. Predicted binding mode of molecular docking between compound **1** and PYGB.

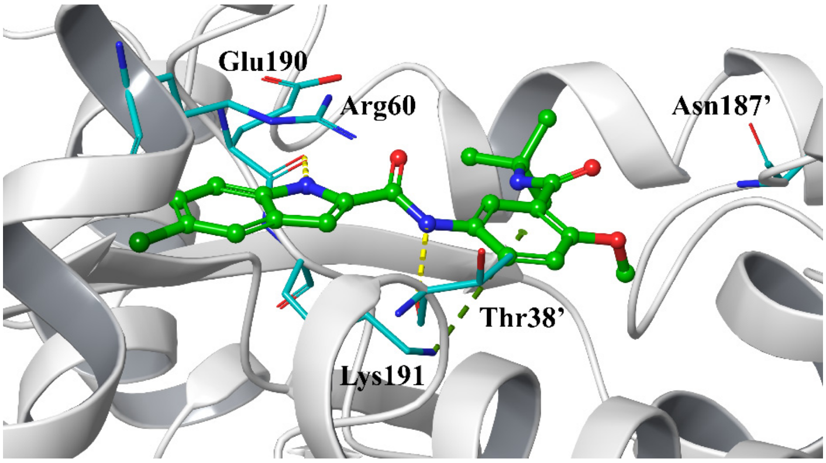

Figure S2. Predicted binding mode of molecular docking between compound **1** and PYGL.

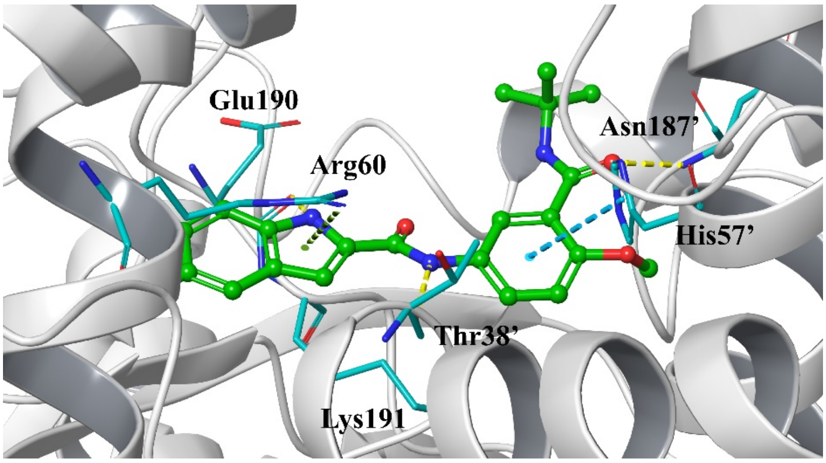

Figure S3. Predicted binding mode of molecular docking between compound **1** and PYGM.

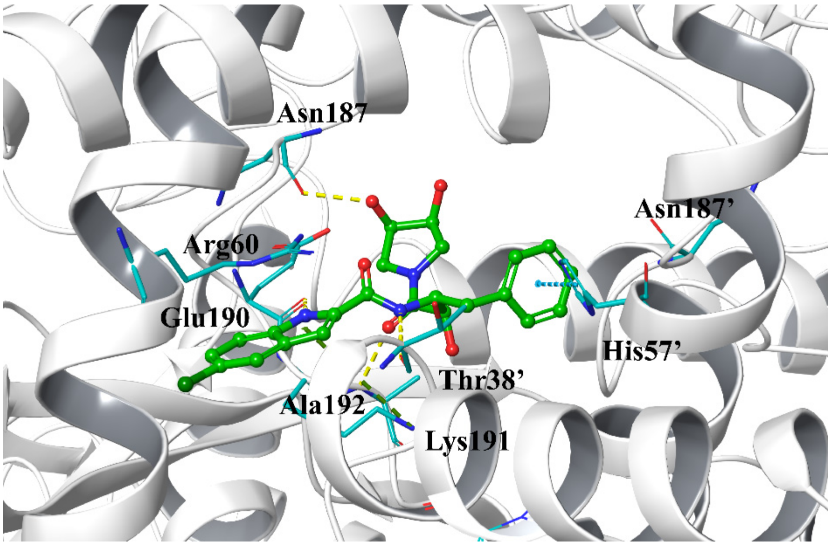

Figure S4. Predicted binding mode of molecular docking between Ingliforib and PYGB.

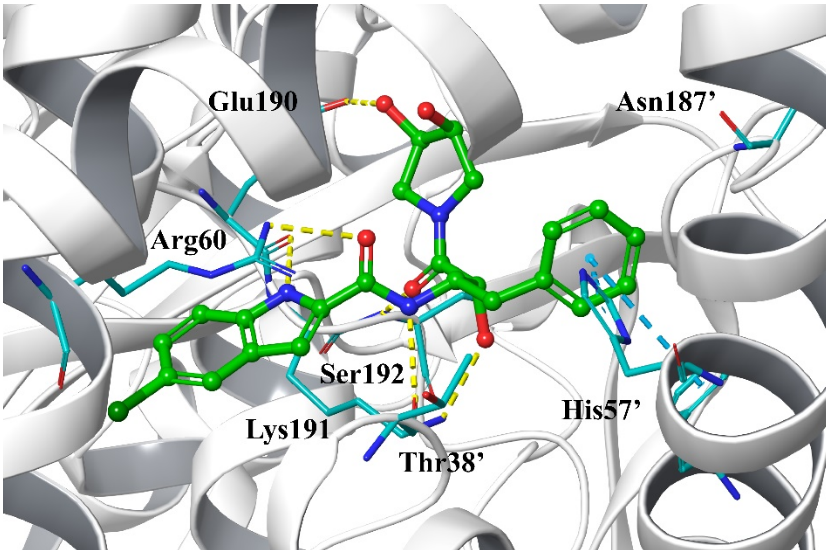

Figure S5. Predicted binding mode of molecular docking between Ingliforib and PYGL.

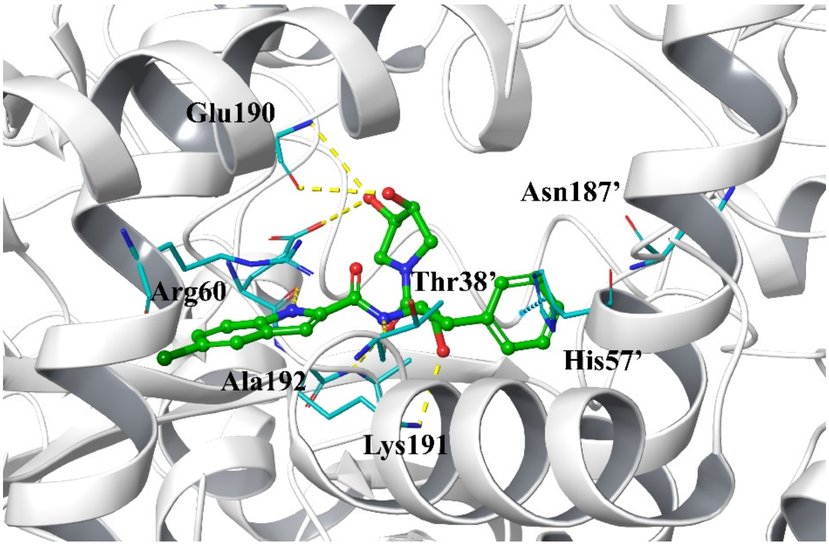

Figure S6. Predicted binding mode of molecular docking between Ingliforib and PYGM.

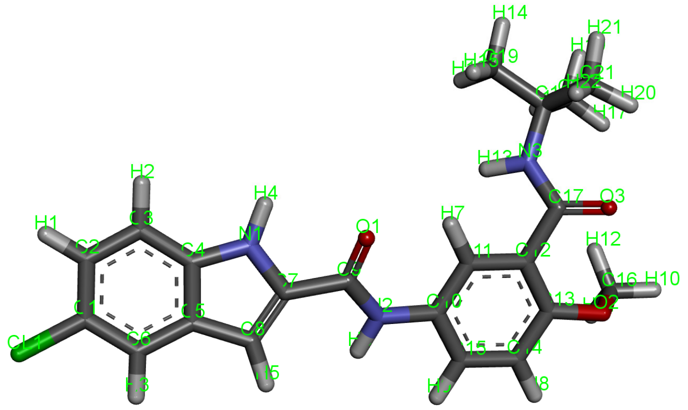

Figure S7. Atomic number of compound **1**.

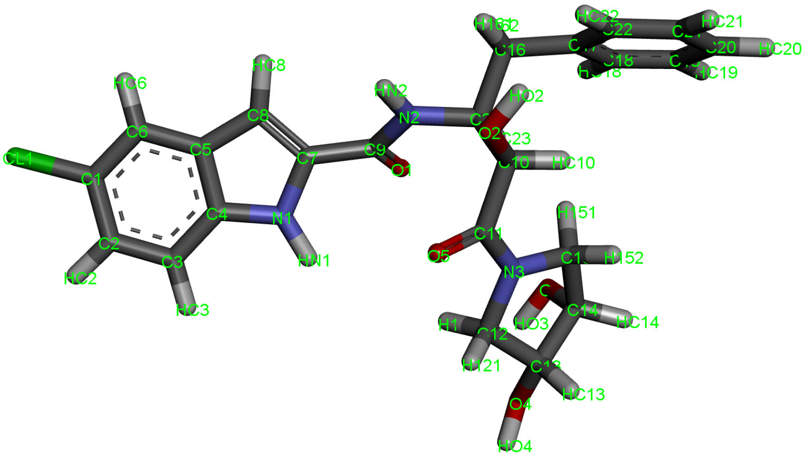

Figure S8. Atomic number of ingliforib.
